# Outpatient care for venous leg ulceration in Germany: A retrospective claims data analysis

**DOI:** 10.1111/ddg.70021

**Published:** 2026-03-12

**Authors:** Lisa Weller, Regina Poß‐Doering, Thomas Grobe, Thomas Fleischhauer, Gunter Laux, Jona Frasch, Attila Altiner, Kerstin Protz, Joachim Szecsenyi, Jonas D. Senft

**Affiliations:** ^1^ aQua Institute for Applied Quality Improvement and Research in Healthcare GmbH Göttingen Germany; ^2^ Department of General Practice and Health Services Research Heidelberg University Hospital Heidelberg University Faculty of Medicine Heidelberg Germany; ^3^ University Medical Center Hamburg‐Eppendorf (UKE) Hamburg Germany; ^4^ Competence Center for Health Services Research in Dermatology (CVderm) and Institute for Health Services Research in Dermatology and Nursing (IVDP) University Medical Center Hamburg‐Eppendorf (UKE) Hamburg Germany

**Keywords:** Chronic wounds, compression therapy, outpatient care, venous leg ulcer

## Abstract

**Background and objectives:**

Outpatient care of venous leg ulcers (VLU) is a problem of high medical and health economic relevance. Treatment is often inconsistent and not guideline‐compliant. The project Ulcus Cruris Care investigated the VLU outpatient care situation in Germany.

**Patients and methods:**

Reimbursement data of 40,046 patient cases (2015–2019) with VLU diagnosis from a statutory health insurer (AOK Baden‐Wuerttemberg, Germany) were retrospectively analyzed. Outcome assessment was based on prescriptions, medical aids, nursing care, use of compression devices, surgical procedures and treatment costs.

**Results:**

47.3% (n  =  7,581) of patients without contraindication received a compression device within 90 days. 2.3% (n  =  135) received surgical truncal vein interventions. 14.1% (n  =  771) received consistent compression therapy associated with faster wound healing (121.6 ± 86.2 vs. 188.1 ± 164.2 days, p < 0.001), lower total costs (4,280 ± 5,326 € vs. 5,683 ± 9,657 €, p < 0.001) and lower disease‐specific costs (2,498 ± 2,785 € vs. 2,797 ± 5,145 €, p  =  0.017) versus no or unsteady treatment.

**Conclusions:**

In outpatient VLU care around 80% of patients do not receive consistent compression therapy and are therefore not adequately treated. There is an urgent need for action to improve treatment of patients with VLU in Germany.

## BACKGROUND

The treatment of chronic wounds represents a major challenge, both medically and economically. Up to 80% of chronic skin ulcerations of the lower extremity can be attributed to chronic venous insufficiency.^1^ The prevalence of venous ulcers in Germany is estimated to be 0.7%–1.4% and increases markedly in individuals aged ≥ 65 years.^2^, ^3^ Due to demographic change, an aggravation of the care problem is to be expected in the future. In addition to the resulting physical, psychological, and social burden of disease on patients, venous leg ulcer (VLU) is also associated with high health economic costs and absenteeism.^2^, ^3^ According to healthcare analyses, treatment costs account for 1% to 2% of the total healthcare budget.^4^

In Germany, the recently revised expert consensus‐based guideline on diagnosis and treatment of venous leg ulcers^1^, ^5^ recommends compression therapy for patients with VLU, provided there are no contraindications present. Compression therapy is an important component of conservative treatment and its effectiveness on wound healing in florid VLU has been clearly proven when applied professionally with a contact pressure in the range of 40–60 mmHg according to evidence class IA.^6^ Its consistent and appropriate application is associated with a lower recurrence rate, a reduction in pain and an improvement in health‐related quality of life.^6^, ^7^ Despite this, compression therapy is only used in around 31%–61% of cases according to health care analyses, and there are deficits in its practical implementation.^8^, ^9^

The Ulcus Cruris Care project aims to develop and implement an evidence‐based, patient‐oriented care concept for the treatment of VLU in general practice on the basis of current guidelines^1^, ^10^, ^11^ and scientific findings. As part of the project, routine billing data from the statutory health insurer AOK Baden‐Wuerttemberg was evaluated to investigate the current VLU outpatient care situation. The present analysis aimed to assess the use of compression therapy as a central treatment element, consultations with physicians, relevant surgical interventions, and overall incurred costs. The findings may support the implementation of appropriate interventions, thereby contributing to improvements in outpatient care for VLU.

## MATERIAL AND METHODS

### Data source

For this retrospective observational study, the statutory health insurer AOK Baden‐Württemberg provided routine billing data on adult patients with at least one outpatient diagnosis of venous leg ulcer (ICD‐10 codes: I83.0, I83.2, I87.01, or I87.21) during the period from 2015 to 2019. The data set comprised insured person master data, outpatient and inpatient care data, information on prescriptions for medications and dressings, prescriptions for remedies and aids, and data on care services. This study was approved by the Ethics Committee of the Medical Faculty of Heidelberg (reference S‐489/2021, 24 June 2021).

### Identification of disease incidence

To identify disease incidence, data on outpatient diagnoses, prescription of bandaging, and standard billing codes in German ambulatory healthcare (EBM) were combined (Figure 1). In the case of VLU, there is usually close contact with doctors with regular prescriptions for bandaging. Cases were therefore determined based on the data available on a daily basis for prescribed bandaging and billing of EBM code “02312” (Treatment complex of one or more chronic venous ulcer(s)). The first date on which either billing code 02312 was charged or a dressing was prescribed was defined as the starting point of an incident disease case, provided that a venous leg ulcer diagnosis was also coded in the outpatient setting in the same quarter. A list of more than 12,000 of the most common products with pharmaceutical central numbers (PZN) was created to identify wound dressings. Affected individuals were classified as incident cases if, following the start date, dressings were prescribed at least once more or EBM code 02312 was billed over a period of at least 56 days. A period of 91 days was selected to differentiate between episodes of illness, during which neither of the two criteria could be documented. Since the evaluation is based on (potential) availability of outpatient data, insured persons with a long hospital stay (> 21 days) within the respective observation period were excluded.

**FIGURE 1:**
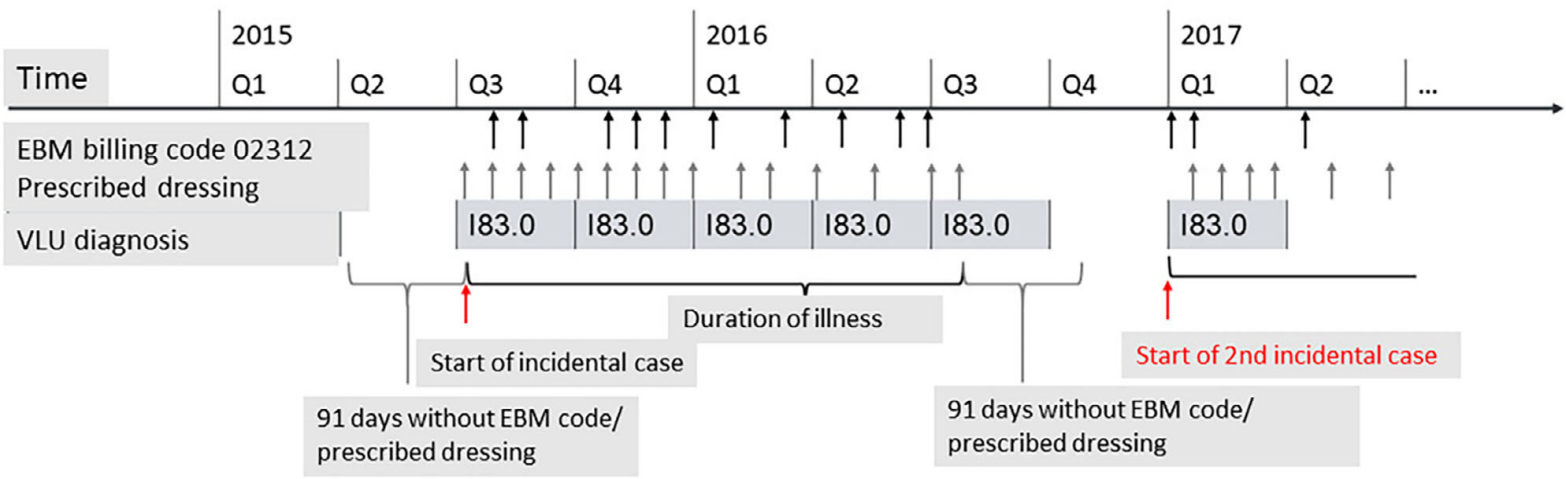
Determination of incident and completed cases of illness based on information on diagnoses, prescribed dressings (VM) and EBM code 02312 (EBM). Each arrow indicates the date of billing for EBM code 02312 (upper row) or a dressing prescription (lower row). The diagnosis of venous leg ulcer (represented in the graph by the ICD‐10 code I83.0) could only be assigned on a quarterly basis and without an exact date. The start of an incident case was defined as either the date of the first billing of EBM code 02312 or the date of the first dressing prescription, provided that a UCV diagnosis was documented in the same quarter. A period of 91 days was used to distinguish between individual episodes of illness. The end of the illness (if determinable) was defined as the date of the last billing of EBM code 02312 or the last dressing prescription, followed by a period of at least 91 days without further prescriptions or billing.

### Outcomes

Treatment with compression therapy was investigated as the primary outcome. Based on medical aid code numbers and a list of more than 1,100 products identified by pharmaceutical central numbers, compression devices were captured, including medical compression stockings, two‐layer ulcer stocking systems, adjustable compression systems, intermittent pneumatic compression, and materials for phlebological compression bandaging (multicomponent systems, short‐stretch bandages, padding materials, and tubular bandages).

Consistent compression therapy based on the routine billing data was defined as the documented prescription of a compression device within 21 days of the start of the case followed by a periodic prescription of a new compression device. A prescription was considered periodic if either a new compression product was prescribed within a specified period of time following prescription of a compression product or the case was considered healed and closed. This period was set at 3 months for compression bandaging and 7 months for all other compression products. Secondary outcomes were frequencies of relevant surgical interventions, disease‐related consultations with general practitioners, and relevant further specialist physicians (surgery, vascular surgery, dermatology, or angiology).

The first 90 days of incident cases were analyzed to assess outcomes. To examine the overall treatment period, outcomes were additionally evaluated for all cases that concluded during the observation period.

### Statistical analysis

Socio‐demographic sample characteristics, and the frequency of compression therapy, surgical interventions, and consultations with general practitioners and other specialist physicians were analyzed descriptively. Wound healing time was calculated for completed cases.

To investigate the effects of consistent compression therapy, cases receiving consistent compression therapy were compared with cases receiving inadequate compression therapy or no compression therapy. Two‐sided t‐tests for independent samples were used for all group comparisons, and frequencies were compared using the χ^2^ test. The significance level was set at p  =  0.05.

### Cost analysis

For a health economic analysis, treatment costs were evaluated from the statutory health insurer perspective and as documented in the billing data. Costs were evaluated for completed cases concluded within the period of illness and in addition, also for a 365‐day period post the first diagnosis, to ensure comparability with prior studies. To this end, for each patient the costs within the specific time frame (i.e., period of illness or 365 days after first diagnosis) were summed up and mean costs were calculated as the sum of all patients’ costs divided by the number of patients. Based on the available case‐specific billing data, costs were determined for outpatient medical care, hospitalization, home nursing care, as well as prescribed medications and medical aids. Specific costs for wound care and compression materials were identified using the PZN and aid identification number. To calculate disease‐specific expenditure, costs for ambulatory care services (general practice, relevant specialist physicians in dermatology, surgery, vascular surgery, and angiology), bandaging, compression material, and ambulatory nursing care were added up.

## RESULTS

### Study population

The initial population available for analysis comprised n  =  40,046 insured adult individuals with at least one diagnosis of venous leg ulcer during the period from 2015 to 2019. A total of n  =  8,092 incident cases were identified, of which 5,782 cases had concluded by the end of the observation period. Table 1 summarizes the characteristics of the study population.

**TABLE 1:** Descriptive statistics of the study population.

	Initial population	Incidental cases	Concluded cases
Number of insured individuals	40,046	8,092	5,782
Age in years (mean ± SD)	71.3 ± 14.5	76.6 ± 12.4	75.6 ± 12.5
Percentage female/male	62.7% / 37.3%	60.8% / 39.2%	60.8% / 39.2%
Individuals with nursing care level at start of illness (%)	–	40.9%	37.2%
Charlson‐Index^1^ at start of illness (mean ± SD)	–	3.4 ± 2.7	3.1 ± 2.6
Individuals with contraindication for compression therapy^2^	–	6.31%	5.38%

*Abbr*.: SD, standard deviation; Charlson Index, Charlson Comorbidity Index

^1^ Accordiung to Glasheen et al.^12^

^2^ Ambulatory diagnosis of decompensated cardiac insufficiency (I50.14, I50.05) or peripheral arterial occlusive disease in Stage IV (I70.24, I70.25)

### Care services for patients with VLU

Of the insured adult individuals with incident disease and no contraindication to compression therapy (outpatient diagnosis of decompensated heart failure [I50.14, I50.05] or stage IV peripheral arterial disease [I70.24, I70.25], n  =  7,581), 47.3% (n  =  3,589) received at least one compression device within the first 90 days after the onset of illness. The first prescription was given on average after 24.3 ± 27.0 days. In 27.5% (n  =  2,084) of the insured individuals, the first compression product was prescribed within the first 21 days. Figure 2 describes the sample sizes and proportions for the provision of compression therapy.

**FIGURE 2:**
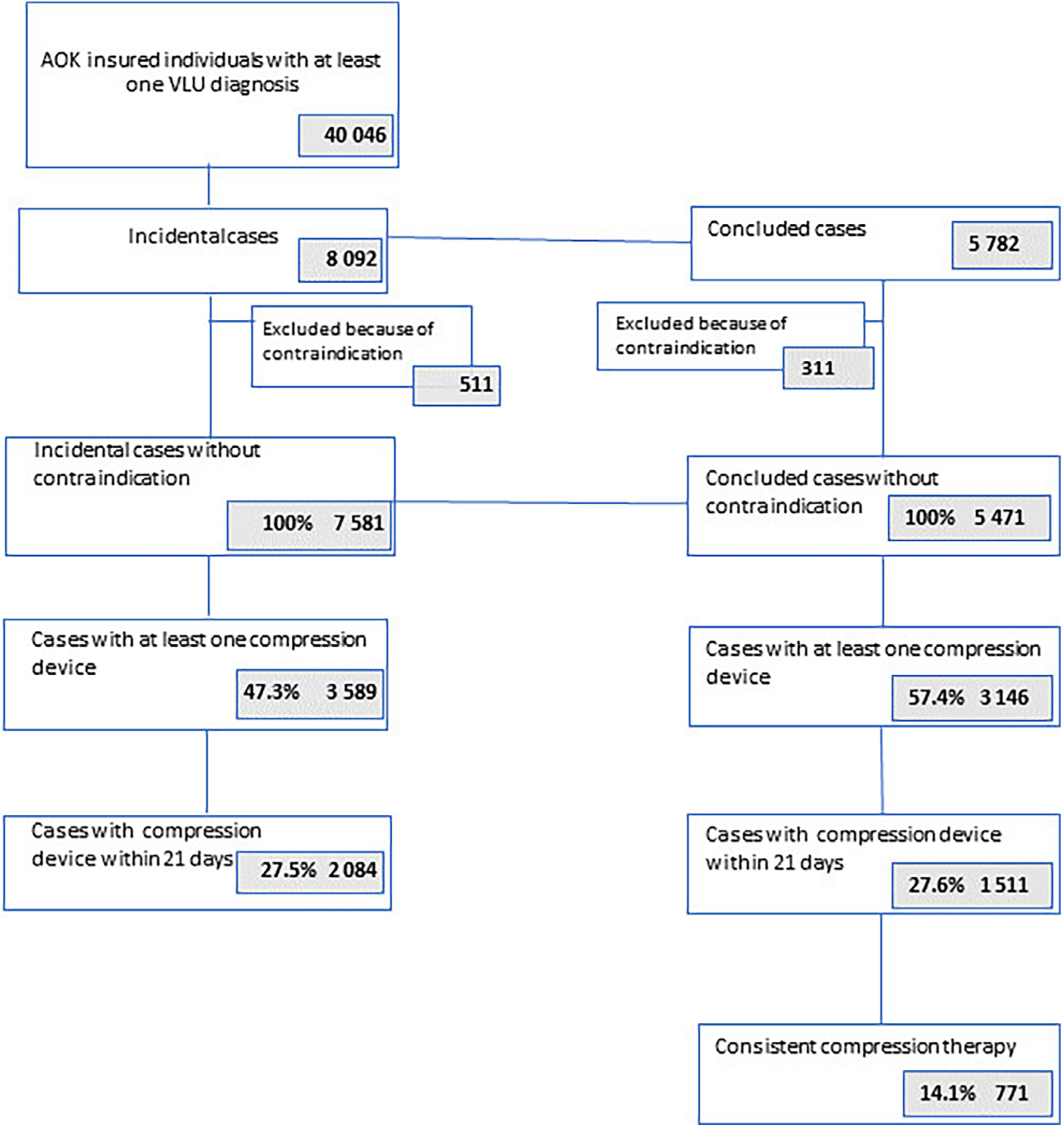
Sample sizes and percentages (%) of patients receiving compression therapy.

Patients without contraindication for whom the presence of VLU from onset to healing fell entirely within the observation period (n  =  5,471), 57.4% (n  =  3,146) received at least one compression product during the illness period. In 27.6% of the cases (n  =  1,511), a compression device was prescribed within the first 21 days. In 14.1% (n  =  771), consistent compression therapy as defined in the study was carried out during the entire period of illness.

In addition, invasive procedures for ablation of varicose veins were performed in n  =  135 of the insured individuals with completed disease (2.3%). In n = 126 of 135 individuals (93.3%), ablation of truncal veins was performed to treat superficial venous insufficiency; among these, 21.5% (n = 29) underwent endovenous ablation. Interventions were performed within the first 90 days after the start of the disease in a total of n  =  94 of the insured persons with incidental disease (1.2%). The majority of the patients with incidental illness were mainly treated by general practitioners (74.2%). More than half (60.9%) were treated exclusively by general practitioners, and 16.6% were mainly treated by specialists. Within 90 days after first diagnosis, patients on average visited a physician six times (SD  =  4.69). Individuals with consistent compression therapy were more frequently predominantly treated by specialists (23.3%) and less frequently predominantly by GPs (64.7%) than patients without consistent compression therapy (predominantly specialists: 16.1%, predominantly GPs: 76.3%; p < 0.001 in each case).

### Wound healing

The average wound healing time for completed cases was 178.9 days ± 157.8 (median: 125). The healing rate within 6 months of the onset of the disease was 69.4%, within 12 months the healing rate was 90.6%. Insured patients who received consistent compression therapy had a shorter wound healing time (121.6 ± 86.2 days) compared to the entire remaining collective (188.1 ± 164.2 days), p < 0.001. The healing rates after 6 months and 12 months were also higher in insured persons with consistent, regular compression therapy (6 months: 85.9%, 12 months: 97.1%) compared to the rest of the group (6 months: 66.7%, 12 months: 85.9%), p < 0.001.

### Cost analysis

Within the duration of illness, the average total costs per patient were € 5,622 ± 9,255 and the disease‐specific costs were € 2,779 ± 4,862. The average annualized total costs per patient were € 8,294 ± 10,805 and the disease‐specific costs were € 3,840 ± 5,566.

The cost comparison between patients with and without consistent compression therapy showed lower total costs (p < 0.001) and lower disease‐specific costs (p  =  0.017) for the duration of the disease for patients with consistent compression therapy (€ 4,280 ± 5,326 and € 2,498 ± 2,785) compared to patients without or with inadequate compression therapy (€ 5,683 ± 9,657 and € 2,797 ± 5,145) with an overall shorter treatment time for patients with consistent compression therapy. When the costs were compared in relation to one year from initial diagnosis, the total costs for both collectives were similar (€ 7,933 ± 10,247 and € 8,019 ± 10,507, p  =  0.84), and the disease‐specific costs were slightly higher for patients with consistent compression therapy than for patients without or with insufficient compression therapy (€ 4,292 ± 4,070 and € 3,682 ± 5,674 respectively, p  =  0.006). A detailed breakdown of the costs for individual areas of the healthcare system is shown in Table 2 (costs within the duration of the illness) and Table 3 (annual costs from the onset of the illness).

**TABLE 2:** Costs in € (median [IQR^0^]) for treating a patient with venous leg ulcer over the duration of disease.

	Total	Individuals without contraindication for compression therapy	
Costs	n = 5,782	Compression therapy^a^ n = 771	No/insufficient compression therapy^b^ n = 4,700
Ambulatory	450 (254–826)	364 (209–617)	460 (263–853)
General practitioners	242 (130–428)	175 (100–312)	252 (136–451)
Specialists^1^	0 (0–89)	0 (0–88)	0 (0–89)
Ambulatory nursing care	0 (0–980)	0 (0–1 150)	0 (0–920)
Prescriptions	1,087 (539–2,160)	1,016 (558–1,899)	1,082 (522–2,197)
Dressing material	436 (198–968)	444 (210–869)	434 (193–984)
Compression devices^2^	45 (0–139)	178 (90–408)	19 (0–104)
Hospital	0 (0–1,297)	0 (0–0)	0 (0–1 240)
Medical aids	76 (0–239)	87 (0–225)	67 (0–234)
Disease‐specific costs	1,502 (730–3,187)	1,689 (901–2,999)	1,446 (696–3,214)
Total costs	2,888 (1,271–6,559)	2,583 (1,330–5,139)	2,864 (1,220–6,603)

^0^ IQR: Interquartile range

^1^ Dermatology, Surgery, Vascular Surgery and Angiology

^2^ Costs for compression devices are partly included in drug prescriptions and partly in medical aids.

^a^ Patients with consequent compression therapy

^b^ Patients without compression devices or with insufficient compression therapy

**TABLE 3:** Costs in € (median [IQR^0^]) for treating an insured patient with venous leg ulcer during the 365‐day period after disease onset.

	Total	Individuals without contraindication for compression therapy	
Costs	n = 5,451	Compression therapy^a^ n = 719	No/insufficient compression therapy^b^ n = 4,448
Ambulatory	1,002 (667–1,495)	964 (665–1,473	1,000 (658–1,485)
General practitioners	532 (388–762)	05 (374–732)	532 (386–762)
Specialists^1^	24 (0–129)	33 (0–138)	20 (0–127)
Ambulatory nursing care	0 (0–1,496)	0 (0–2,440)	0 (0–1,316)
Prescriptions	1,958 (1,056–3,275)	1,954 (1,043–3,286)	1,921 (1,034–3,210)
Dressing material	514 (239–1,050)	506 (256–999)	509 (230–1,062)
Compression devices^2^	52 (0–186)	264 (132–610)	0 (0–139)
Hospital	0 (0–1,546)	0 (0–0)	0 (0–1,426)
Medical aids	105 (0–486)	159 (0–545)	103 (0–466)
Disease‐specific costs	2,413 (1,429–4,915)	3,032 (1,696–5,674)	2,308 (1,379–4,705)
Total costs	5,202 (2,735–10,243)	5,264 (2,861–9,528)	5,044 (2,634–10,033)

^0^ IQR: Interquartile range

^1^ Dermatology, surgery, vascular surgery and angiology

^2^ Costs for compression devices are partly included in drug prescriptions and partly in medical aids.

^a^ Patients with consequent compression therapy

^b^ Patients without compression devices or with insufficient compression therapy

## DISCUSSION

### Care services for patients with VLU

The aim of this study was to describe the current care situation of adult individuals with VLU, particularly with regard to the use of causally oriented treatment approaches such as the implementation of phlebological compression therapy or surgical procedures to treat the underlying venous hypertension. The results suggest that phlebological compression therapy is not at all or not consistently implemented in VLU care, even in the absence of contraindications. Less than half of the patients received a compression device within the first 90 days of the disease. Less than one in five received consistent, regular and early compression therapy. Invasive truncal vein ablation as a further causal therapeutic approach to treat insufficient superficial veins was only received by around 2% of the patients. Overall, the data thus clearly show that patients with VLU were not adequately treated.

With regard to the use of compression devices in the group of VLU patients, the results are in line with older care analyses, which showed use rates of around 40%.^8^, ^9^ However, the modeling of the disease progression in this analysis also suggests that, in contrast to current guideline recommendations, compression therapy is only used early and consistently in very few cases.^1^ Furthermore, invasive procedures like ablation of truncal veins for treatment of superficial vein disease are rarely performed in Germany. Therefore, based on the analyzed data, it must be assumed that more than 80% of patients do not receive sufficient causally oriented treatment. Consequently, there is an acute need to implement current guideline recommendations in the care of patients with VLU. Studies show that consistent compression therapy leads to a shortening of wound healing time and a reduction in the recurrence rate.^6^, ^7^, ^12^ This is confirmed by the evaluation presented here: VLU patients with consistent, regular, and early compression therapy had a shorter wound duration than patients who did not.

The reasons for inconsistent or absent compression therapy are multifactorial. One reason for the low rate of compression therapy may be a lack of knowledge about the relevance of compression therapy among healthcare providers.^13^, ^14^ However, inadequate patient education also contributes to compression therapy not being used or not being continued.^15^, ^16^ In addition, the involvement of many different stakeholders may also lead to a lack of consistent compression therapy and a tendency for patients to take a more passive role.^14^ Patient‐centered approaches, especially the use of education for patients with VLU, have an impact on adherence and wound healing and should therefore be considered as an integral part of outpatient chronic wound care.^17^, ^18^ As confirmed in this study, the vast majority of patients with venous leg ulcers regularly present to their general practitioner. Therefore, the implementation of potential interventions – such as increasing professional competence and promoting patient‐centered care – appears particularly relevant in the primary care setting. In addition, the competence to determine the indication and practical implementation of compression therapy should be promoted among all those involved in chronic wound care. Future comparative studies may be useful to assess efficacy of primary care‐led, specialist care‐led, and combined care models. There is a particular need to optimize correct practical implementation, for example by outpatient nursing care services.^19^ Eventually, in order to specifically promote the use of compression therapy in line with the guidelines, further studies will be needed to investigate the existing barriers to its implementation.

### Cost analysis

For this present study, costs of VLU disease were calculated from the perspective of statutory health insurance. The average total patient‐related costs were around € 5,600 during the period of illness or around € 8,300 per year. These figures are therefore higher than those reported in previous studies, which estimated total annual costs of approximately € 4,000.^20^, ^21^ An advantage of the cost calculation approach used here, which accounts for the individual duration of disease, is that venous leg ulcer‐specific costs can be estimated more accurately than in previous studies. However, this approach also has the disadvantage that inpatient costs cannot be adequately calculated, as patients with long hospital stays had to be excluded for methodological reasons in order to determine the duration of illness. Patients with consistent, regular, and early compression therapy showed lower total costs and lower disease‐specific costs over the duration of the disease than the other patients. In the 12‐month analysis, the initial cost reduction was not sustained, most likely due to a wash‐out effect driven by overall health care costs and comorbidities.

Nevertheless, the results indicate that compression therapy can contribute to cost savings through faster wound healing, which seems plausible. This also applies to indirect costs, such as those arising from days of work incapacity, which could not be captured in this analysis. In addition, substantial cost savings are likely attributable to a reduction in the need for dressings, which accounted for a considerable proportion of disease‐specific costs (just under € 1,000). This aspect is of particular relevance as the incidence of chronic wounds is expected to rise sharply over the next 10–20 years due to demographic trends.^22^

### Limitations

With regard to the findings of the present study, it should be noted that the underlying data were generated for billing purposes. Claims data lack several specific disease parameters, such as classification or wound size. Therefore, it cannot be determined whether patients with larger or more complex wounds are more frequently treated with compression therapy. Such a bias may also explain why cost differences between cases with consistent compression therapy and the remaining cohort are no longer detectable after one year. Another important limitation inherent to claims data is underreporting. This concerns both the identification of VLU cases via ICD‐10 codes, as performed in this study, and the determination of disease‐specific costs. Not all costs can be clearly attributed to the disease, as disease‐specific billing items are often missing, or ‐ such as the EBM code 02312 ‐ are used too rarely. Such distortions should be similar for cases with and without consistent compression therapy and thus still allow comparisons between the groups. Costs for dressings and compression products can be allocated relatively specifically to the illness, and might only be incorrectly allocated in individual cases. With regard to invasive procedures to treat superficial vein reflux, it has to be considered that endovenous ablations are not regularly reimbursed by statutory health insurance in Germany, therefore the numbers may be underestimated in this regard.

## Conclusions

The present analysis shows that in outpatient care for venous leg ulcers, approximately 80% of patients receive neither consistent compression therapy nor surgical treatment and are therefore inadequately treated. There is an urgent need for action to improve treatment of patients with VLU in primary care in Germany. Proposed solutions should aim to enhance the competence of all stakeholders involved and to foster patient‐centered treatment approaches, particularly in primary and outpatient care. There is an urgent need to shift awareness away from a primary focus on local wound management toward comprehensive, causal treatment of venous leg ulcers. A consistent implementation of causally oriented treatment could avert long‐term chronification of the wound situation and significantly improve the quality of life for many affected patients.

## FUNDING

The Ulcus Cruris Care project is funded by the Innovation Committee of the Joint Federal Committee G‐BA ((01VSF19043).

## CONFLICT OF INTEREST STATEMENT

K.P. received honoraria for consulting and/or lectures from Bauerfeind, Essity/Jobst, Curea, Hartmann, Lohmann & Rauscher, Juzo, medi, and Urgo. All other authors declare no conflict of interest.
